# Clinical Effects of Korean Red Ginseng in Postmenopausal Women With Hand Osteoarthritis: A Double-Blind, Randomized Controlled Trial

**DOI:** 10.3389/fphar.2021.745568

**Published:** 2021-11-08

**Authors:** Hye In Kim, Seung Joo Chon, Ki Eun Seon, Seok Kyo Seo, Yun-Rak Choi

**Affiliations:** ^1^ Department of Obstetrics and Gynecology, Severance Hospital, Yonsei University College of Medicine, Seoul, South Korea; ^2^ Department of Obstetrics and Gynecology, Gil Hospital, Graduate School of Medicine, Gachon University of Medicine and Science, Inchon, South Korea; ^3^ Department of Orthopedic Surgery, Severance Hospital, Yonsei University College of Medicine, Seoul, South Korea

**Keywords:** cartilage, osteoarthritis, pain, panax ginseng, postmenopausal state, randomized controlled trial

## Abstract

**Background:** Although many menopausal Asian women use herbal remedies for joint pain, there are no studies evaluating the efficacy of Korean red ginseng on osteoarthritis symptoms in postmenopausal women. The purpose of this study is to analyze antioxidant enzyme activity, oxidative stress markers, and pain scores before and after red ginseng consumption, to assess its effect in postmenopausal women. Methods. This prospective, double-blind, randomized controlled trial enrolled 52 postmenopausal women who presented with hand edema and/or pain and were diagnosed as degenerative arthritis of the hand. Patients were randomly assigned to the red ginseng (RG) group (supplemented with 3 g/d of RG for 12 weeks) or the placebo group. Changes in pain and Disability of the Arm, Shoulder, and Hand (DASH) scores, antioxidant enzyme, oxidative stress markers, serum estradiol levels, and endometrial thickness were analyzed. Results. The pain score and DASH score were significantly improved in the RG group (both *p* < 0.05). The improvement of pain score at rest, during work or sport, and DASH score was significant compared to that of the placebo group. The superoxide dismutase level increased (*p* < 0.05) and the malondialdehyde level decreased (*p* < 0.05) significantly in the RG group, while none of the antioxidative factors showed a significant change in the placebo group. Serum estradiol levels and endometrial thickness were not affected by RG supplementation. Conclusion. RG may be an effective dietary supplement for postmenopausal women with degenerative osteoarthritis of the hand. It may relieve pain and improve antioxidative activity without the risk of endometrial thickening.

## Introduction

Many women experience vasomotor symptoms such as hot flushes and night sweats during the perimenopausal period. However, the prevalence of such symptoms and the perception of their discomfort are influenced by several different factors, including ethnicity, biological environment, lifestyle, overall health, and socioeconomic status (Obermeyer, 2000). There is evidence that the prevalence of menopausal symptoms is different in Asian women compared to that in Western women. Asian women suffer less from vasomotor symptoms but more commonly report joint pain (Mccarthy, 1994; Hilditch et al., 1999; Haines et al., 2005).

Some observational studies have reported favorable effects of exogenous estrogen on joint pain (Nevitt et al., 1996; Wluka et al., 2001; Szoeke et al., 2006; Szoeke et al., 2008). In addition, post hoc analyses of the Women’s Health Initiative (WHI) randomized controlled trial (RCT) have also shown that the use of estrogen alone significantly reduced joint pain in postmenopausal women (Chlebowski et al., 2018). Based on such results, hormone therapy may help alleviate joint pain. However, many women use herbal remedies instead of hormone therapy because of concerns about possible adverse effects of long-term hormone therapy.

Ginseng root (Panax ginseng C.A. Meyer) has been widely used in East Asian traditional medicine to improve general health and treat various conditions. Ginsenosides are the major constituent of ginseng root and exhibit a large variety of biological and pharmacological activities. Red ginseng (RG) is manufactured by steaming and drying fresh white ginseng (WG) and contains newly identified ginsenosides, which are believed to have more potent pharmacological activities than those of WG (Kim et al., 2000). Experimental studies have demonstrated that certain ginsenosides have the potential to be used as therapeutic agents in patients with osteoarthritis (OA) (Cheng et al., 2013; So et al., 2013; Lee et al., 2014). However, no clinical studies have investigated the effects of RG as an alternative therapy for OA symptoms in postmenopausal women.

A previous study has shown that RG did not relieve the vasomotor symptoms but did reduce the Kupperman index and Menopause Rating Scale scores (Kim et al., 2012). In addition, RG was demonstrated to have antioxidant effects, which may be of some benefit in preventing the destruction of articular cartilage as a result of oxidative stress (Seo et al., 2014). However, this study did not focus on whether RG can help relieve OA symptoms. This study was conducted to evaluate the effect of RG on joint pain related to OA in postmenopausal women in various circumstances. In addition, we assessed the cartilage markers such as cartilage oligomeric matrix protein (COMP) and C-terminal crosslinked telopeptide type II collagen (CTXII), oxidative stress, and hormone levels before and after RG or placebo consumption.

## Materials and Methods

### Participants

This study recruited participants from among patients who visited the outpatient clinic of orthopedic surgery with the chief complaints of pain and edema of the hand and were diagnosed with degenerative OA by x-ray. All the patients were asked if they were menopaused and only the patients who were confirmed to be menopaused were asked to enroll to the study. Body measurements (height, weight, body mass index (BMI), blood pressure) and lipid profiles were obtained for all the study participants.

Patients with a diagnosis of rheumatoid arthritis, traumatic arthritis, or other orthopedic diseases such as rotator cuff tear, frozen shoulder, trauma, tenosynovitis, or peripheral neuritis were excluded because these diseases could affect the pain and function of the hand. Patients with chronic anti-inflammatory analgesic use for more than 1 month for degenerative arthritis were also excluded.

### Study Design

This study was a single-center, double-blind RCT. After the initial screening visit and examination, all participants were allocated to either the RG or placebo group in a 1:1 ratio using a computer-generated random number sequence by Biostatistics Collaboration Unit (BCU) of Yonsei college of medicine. The random number sequence was kept secret to both investigators and participants until the end of trial to both investigators and participants. The permutation of random numbers generated by the SAS^®^ system’s Randomization program. The randomization table was devised and generated before clinical trials through SAS^®^.

RG and placebo capsules were provided by the Korea Ginseng Corporation (Daejeon, Korea) for the trial. The company packaged the tablets with the label according to the randomization number and supplied them to the testing institution before the trial. For double-blindness, the investigator provided the capsule with unique code consistent with the allocation number of the participants.

The RG group received 1 g of RG three times daily while the placebo group received identically shaped capsules composed of 95.25% cornstarch, 4% ginseng aromatic powder, 0.15% natural dye, and 0.6% caramel dye, to be taken three times a day for 12 weeks. Each RG capsule contained 500 mg of RG.

The ginsenoside composition in the RG was analyzed by high-performance liquid chromatography. 1 g of ginseng dried sample pulverized by 80–100 mesh was weighed in a centrifugation tube. 10 ml 50% MeOH was added and mixed uniformly. It was cooled for 1 h at 80°C and centrifugation (3,000 rpm, 10 min) was performed. The supernatant was taken. This process was repeated. The supernatant was vacuum-concentrated in a 50°C water tank using a rotary evaporator. The concentrate was dissolved in 2 ml of distilled water filtered with a 0.45 μm membrane filter. It was analyzed by a validated method of high-performance liquid chromatography. According to the standard method required by Health Functional Food Acts of South Korea, HPLC analysis was performed with Halo^®^ RP-Amide column (4.6 × 150 mm, 2.7 μm, Advanced Materials Technology, Inc., DE, United States) at 50°C (Fuzzati, 2004). Mobile phase was 0–6 min: 27–28% acetonitrile; 6–10 min: 28%; 10–30 min: 28–34%; 30–33 min: 34–80%; 33–35 min: 27% acetonitrile and gradient eluted. The flow rate was 0.5–0.8 ml/min and the absorbance was measured at the 203 nm wavelength of the UV detector . It was found to contain Rg1 (2.61 mg/g), Rb1 (4.26 mg/g), Rb2 (1.65 mg/g), Rg2s (0.20 mg/g), Rg3s (0.13 mg/g), Rc (1.80 mg/g), Rd (0.29 mg/g), Re (1.71 mg/g), Rf (0.67 mg/g), and Rh1 (0.11 mg/g).

For the HPLC analysis, reference standard method was used. Regarding the precision of the HPLC, the results and statistical values of the three repeated tests by adjusting the amount of ginsenoside Rg1, Rb1 and Rg3s to 0.25, 0.5, and 0.75 g was presented in Table 1. Regarding the accuracy and percentage recovery, we added the saponin fraction corresponding to 100%, 150%, and 200% of the amount of ginsenoside Rg1, Rb1, and Rg3s present in 0.5 g of the sample and the test was repeated 3 times. The statistical values and recovery rates was presented in Table 2. The lower limits of detection (LLOD) and lower limits of quantification (LLOQ) of Rg1, Rb1 and Rg3s was presented in Table 3. All the other information about the HPLC analysis was presented in Supplementary Material.

**TABLE 1:** Precision of HPLC analysis.

	Rg1		Rb1		Rg3s	
	Mean ± SD	Precision (%RSD)	Mean ± SD	Precision (%RSD)	Mean ± SD	Precision (%RSD)
Level 1 (0.25 g)	2.808 ± 0.009	0.33	4.994 ± 0.020	0.39	0.228 ± 0.004	1.60
Level 2 (0.5 g)	2.697 ± 0.073	2.70	4.758 ± 0.123	4.65	0.211 ± 0.005	2.13
Level 3 (0.75 g)	2.654 ± 0.007	0.25	4.641 ± 0.012	0.26	0.211 ± 0.003	1.47
Between-level	2.720 ± 0.038	1.38	4.798 ± 0.062	1.30	0.217 ± 0.001	0.32

SD, standard deviation; SD, standard deviation; RSD, releative standard deviation.

**TABLE 2:** Accuracy and percentage recoveray of HPLC analysis.

	100% (Mean ± SD)	150% (Mean ± SD)	200% (Mean ± SD)
Rg1	100.0 ± 0.40	101.9 ± 0.87	100.8 ± 1.03
Rb1	99.9 ± 0.55	101.7 ± 0.51	101.0 ± 1.40
Rg3s	91.8 ± 1.36	95.4 ± 0.82	97.5 ± 1.96

SD, standard deviation; SD, standard deviation.

**TABLE 3:** LLOD and LLOQ of HPLC analysis.

Components	LLOD(μg/mL)	LLOQ(μg/mL)
Rg1	1.74	5.8
Rb1	0.89	2.87
Rg3s	0.34	1.12

LLOD, lower limits of detection; LLOQ, lower limits of quantification.

### Measurements

The pain score was obtained *via* self-report questionnaire. The participants were asked to score the pain in certain circumstances according to Visual Analog Scale (VAS) at the baseline (week 0) and final (week 12) visits. The Disabilities of the Arm, Shoulder and Hand (DASH) score was also assessed by a self-reported questionnaire (Hudak et al., 1996; Williams, 2013). The questionnaire consists of 30 items among which 21 questions evaluate the difficulty of a specific task, 5 questions evaluate symptoms, and 4 questions evaluate social function, work function, sleep, and confidence. The DASH score range is from 0 to 100, and the higher the score, the higher the upper limb disability. Anthropometric measurements were obtained, and blood was drawn for laboratory testing at the baseline (week 0) and final (week 12) visits. Body weight and height were measured with the participants in light indoor clothing with InBody analyzer (Inbody Co., Ltd., Seoul, South Korea) that also calculated BMI as the weight divided by the height squared (kg/m2). Blood pressure was measured with the participant in the sitting position, after 5 min of rest, using an automated device (TM2665P; A&D Co., Ltd., Tokyo, Japan). Blood samples were collected in sterile tubes from an antecubital vein and were centrifuged at 300 g for 10 min. The serum samples were stored at -80.1°C until analysis.

Enzyme-linked immunosorbent assays were performed using various commercial kits. Serum superoxide dismutase (SOD) (Cayman Chemical Company, Ann Arbor, MI, United States) was measured to assess antioxidative enzyme activity. Malondialdehyde (MDA) was measured as an oxidative stress marker (Cell Biolabs Inc., San Diego, CA, United States). COMP (Kamiya biomedical company, Tukwila, WA, United States) and urinary CTXII (UCSN Life Sciences, Inc., Wuhan, China) were also measured.

### Statistical Analysis

Data were analyzed by intention-to-treat analysis and expressed as mean ± SD. Primary validation variables included those related to pain and function: the pain score in VAS and the DASH score. The secondary validation variables were cartilage damage index, antioxidant enzyme activity, and oxidative stress markers. A paired *t*-test was used to compare the mean changes from baseline to 12 weeks within each group, and a Two sample *t*-test was used to compare the RG and placebo groups. Statistical analyses were performed using Statistical Package for the Social Sciences (SPSS) 15.0 software (SPSS Inc., Chicago, IL, United States). *p*-values ≤0.05 were considered statistically significant.

### Ethical Approval

This study was approved by the institutional review board of Severance Hospital (IRB No. 4-2013-0713) and performed in accordance with the principles of the Declaration of Helsinki. Written informed consent was obtained from all participants.

This trial has been registered with the Clinical Research Information Service [CRIS, (http://cris.nih.go.kr)], Republic of Korea (KCT0006326).

## Results

**FIGURE 1:**
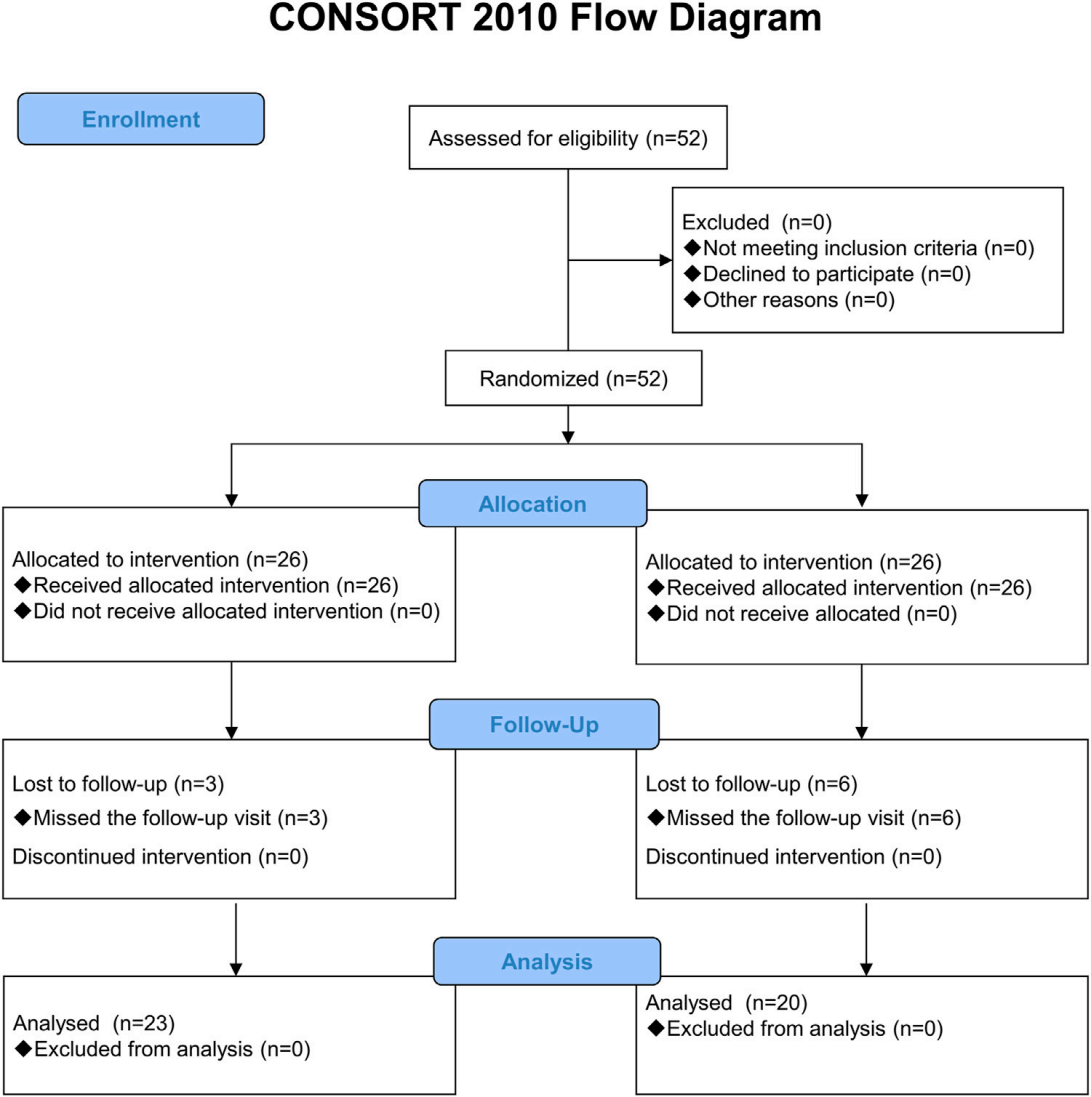
Study flow chart.

A total of 52 participants were enrolled, with 26 participants randomly assigned to each study group (Figure 1). There were three participants in the RG group and six in the placebo group who dropped out of the study and failed to attend the follow-up session. Table 4 shows the baseline characteristics of the participants. There was no significant difference between the two groups in their age, height, weight, BMI, systolic and diastolic blood pressure, pain scores at rest, daily activities, physical activity at work or sports, baseline DASH scores, and the laboratory variables such as SOD and MDA.

**TABLE 4:** Baseline demographic and clinical characteristics of participating postmenopausal women.

Variables	RG group (N = 23)	Placebo group (N = 20)	*p*-value
Age (y)	60.17 ± 9.49	60.55 ± 7.86	0.889
Height (cm)	154.90 ± 4.30	156.13 ± 5.14	0.466
Weight (kg)	56.64 ± 7.90	58.47 ± 6.17	0.477
BMI (kg/m^2^)	23.58 ± 2.98	23.99 ± 2.47	0.675
Systolic blood pressure (mmHg)	127.29 ± 11.63	125.53 ± 7.72	0.623
Diastolic blood pressure (mmHg)	77.17 ± 8.15	74.93 ± 8.37	0.449
Pain score at rest	2.52 ± 1.24	3.30 ± 1.59	0.208
Pain score at daily activities	4.70 ± 1.64	4.85 ± 1.57	0.754
Pain score at work or sports	6.00 ± 1.62	6.20 ± 2.07	0.794
DASH score	38.34 ± 15.58	36.47 ± 17.74	0.715
SOD (U/mL)	266.11 ± 35.60	280.11 ± 42.01	0.243
MDA (nmol/mg)	235.89 ± 46.25	204.89 ± 41.81	0.421
Estradiol (pg/ml)	11.17 ± 5.63	11.16 ± 5.50	0.991
Endometrial thickness (mm)	0.30 ± 0.09	0.28 ± 0.09	0.618

RG, red ginseng; BMI, body mass index; DASH, disabilities of arm, Shoulder and Hand; SOD, superoxide dismutase; MDA, malondialdehyde.

Table 5 shows the pain scores in various circumstances and DASH scores for both groups at baseline and after 12 weeks of treatment. The pain score at rest for the RG group decreased significantly after treatment (*p* < 0.05), and the improvement was statistically significant compared to that of the placebo group (*p* < 0.05). The pain score at various circumstance were all significantly improved in RG group after 12 weeks of red ginseng consumption (*p* < 0.05). Even though the pain score at rest, during daily activity and during work or sport was also improved in placebo group (*p* < 0.05), the change from baseline to week 12 were significantly different between RG group and placebo group in all variables except for the pain score during daily activity (*p* < 0.05 and *p* = 0.49, respectively). After RG supplementation, the DASH score decreased by approximately 26% in the RG group (*p* < 0.05), but this change was not statistically significant compared with that of the placebo group (*p* = 0.37).

**TABLE 5:** Pain score and disability of the arm, shoulder, and hand (DASH) scores at baseline and week 12.

	RG group (N = 23)	Placebo group (N = 20)	*p*-value^a^
Pain score at rest	Baseline: 2.52 ± 1.24	Baseline: 3.30 ± 1.59	0.040
	Week 12: 1.04 ± 0.88	Week 12: 2.50 ± 1.57	
	*p*-value^b^: <0.001	*p*-value^b^: 0.006	
Pain score at daily activities	Baseline: 4.70 ± 1.64	Baseline: 4.85 ± 1.57	0.486
	Week 12: 4.30 ± 1.10	Week 12: 3.75 ± 2.02	
	*p*-value^b^: <0.001	*p*-value^b^: 0.012	
Pain score at work or sports	Baseline: 6.00 ± 1.62	Baseline: 6.20 ± 2.07	0.023
	Week 12: 3.87 ± 1.89	Week 12: 5.15 ± 2.13	
	*p*-value^b^: <0.001	*p*-value^b^: 0.006	
DASH score	Baseline: 38.34 ± 15.58	Baseline: 36.47 ± 17.74	0.021
	Week 12: 20.87 ± 12.56	Week 12: 30.33 ± 17.69	
	*p*-value^b^: <0.001	*p*-value^b^: 0.139	

RG, red ginseng; DASH, disabilities of arm, shoulder and hand.

b Paired *t*-test comparison between means at baseline and at week 12 within groups.

a Comparison of supplementation effects by comparing the change from baseline to week 12 between groups by two-sample *t*-test.

Table 6 shows the change in antioxidant enzyme activity and oxidative stress markers before and after the 12 weeks of treatment. After 12 weeks of treatment, the SOD level increased, and the MDA level decreased significantly in the RG group (both *p* < 0.05), while these levels did not change significantly in the placebo group (*p* = 0.09, *p* = 0.33, respectively). However, the changes in SOD and MDA levels were not significantly different between the two groups (*p* = 0.19, *p* = 0.32, respectively). The oxidized low-density lipoprotein (oxLDL) level did not change in either group after 12 weeks of treatment (*p* = 0.46, RG group; *p* = 0.29, placebo group).

**TABLE 6:** Antioxidant enzyme activity and oxidative stress markers at baseline and week 12.

	RG group (N = 23)	Placebo group (N = 20)	*p*-value^a^
SOD (U/mL)	Baseline: 266.11 ± 35.60	Baseline: 280.11 ± 42.01	0.190
	Week 12: 312.10 ± 51.40	Week 12: 301.95 ± 38.04	
	*p*-value^b^: 0.002	*p*-value^b^: 0.095	
oxLDL (U/L)	Baseline: 31.54 ± 14.58	Baseline: 33.03 ± 17.54	0.431
	Week 12: 33.07 ± 0.10	Week 12: 36.31 ± 18.99	
	*p*-value^b^: 0.458	*p*-value^b^: 0.285	
MDA (nmol/mg)	Baseline: 215.89 ± 46.25	Baseline: 204.89 ± 41.82	0.322
	Week 12: 182.08 ± 50.51	Week 12: 190.65 ± 50.20	
	*p*-value^b^: 0.018	*p*-value^b^: 0.333	

RG, red ginseng; SOD, superoxide dismutase; oxLDL, oxidized low-density lipoprotein; MDA, malondialdehyde.

b Paired *t*-test comparison between means at baseline and at week 12 by group.

a Comparison of supplementation effects between two groups by two-sample *t*-test.

Table 7 shows the changes in estradiol level and endometrial thickness after the treatment. In both groups, the estradiol level did not change after treatment (*p* = 0.63, RG group; *p* = 0.28, placebo group). Moreover, the endometrial thickness, which has been well documented to be affected by hormone levels, did not increase in either group (*p* = 0.71, RG group; *p* = 0.43, placebo group).

**TABLE 7:** Serum estradiol levels and endometrial thickness at baseline and week 12.

	RG group (N = 23)	Placebo group (N = 20)	*p*-value^a^
Estradiol (pg/ml)	Baseline: 11.17 ± 5.62	Baseline: 11.16 ± 5.50	0.569
	Week 12: 10.72 ± 5.71	Week 12: 9.86 ± 6.27	
	*p*-value^b^: 0.634	*p*-value^b^: 0.278	
Endometrial thickness (mm)	Baseline: 0.30 ± 0.09	Baseline: 0.28 ± 0.09	0.447
	Week 12: 0.29 ± 0.10	Week 12: 0.30 ± 0.08	
	*p*-value^b^: 0.709	*p*-value^b^: 0.430	

RG, red ginseng.

b Paired *t*-test comparison between means at baseline and at week 12 by group.

a Comparison of supplementation effects between two groups by two-sample *t*-test.

**FIGURE 2:**
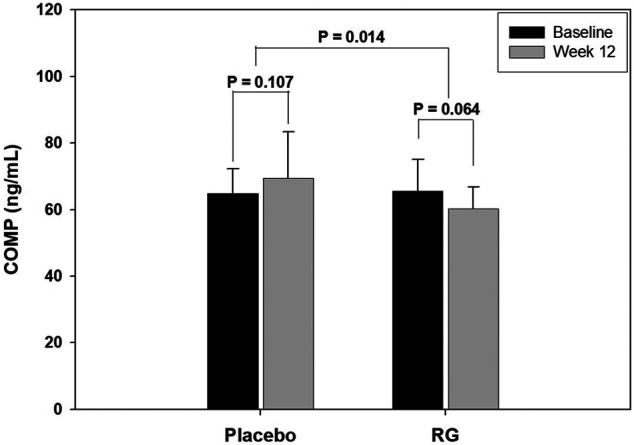
Change in cartilage oligomeric matrix protein (COMP) level after 12 weeks of treatment.

**FIGURE 3:**
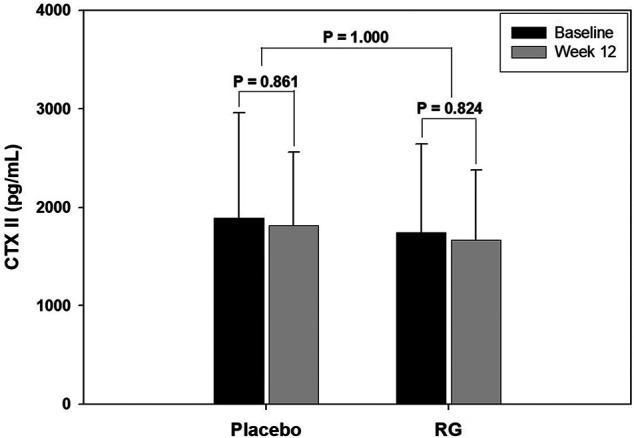
Change in crosslinked telopeptide type II collagen (CTXII) level after 12 weeks of treatment.

Figure 2 shows the change in COMP serum level in each group after 12 weeks of treatment. COMP is well accepted as a diagnostic as well as prognostic indicator of OA (Tseng et al., 2009). After 12 weeks of treatment, the COMP level of the RG group decreased, but the change was not statistically significant (*p* = 0.06). However, the COMP level in the placebo group increased without statistical significance (*p* = 0.11), and the change after 12 weeks was significantly different between the two groups (*p* = 0.01). Figure 3 presents the CTXII levels of the two groups after 12 weeks of treatment. CTXII is another marker of cartilage degradation. The change in both groups was not statistically significant after treatment (*p* = 0.82, RG group; *p* = 0.86, placebo group).

## Discussion

This study evaluated the effect of RG supplementation on OA pain, cartilage markers, oxidative stress, and hormone levels in postmenopausal women. The 12-weeks RG treatment improved the pain score at rest, during daily activity and work or sport, and the DASH score in the RG group. The improvement in pain score at rest, during work or sport and DASH score was statistically significant compared to that of the placebo group. Further, SOD levels increased, and MDA levels deceased significantly in the RG group after the treatment, indicating a reduction in oxidative stress. Lastly, the COMP level, one of the cartilage degradation markers, showed a decrease in the RG group after 12 weeks of treatment, although this was not statistically significant.

In 2002, the WHI reported the effects of combined conjugated equine estrogens (CEE) and medroxyprogesterone acetate in postmenopausal women in their first report about hormone replacement therapy (HRT), and their later report compared the effects of CEE monotherapy compared to placebo (Rossouw et al., 2002; Stefanick et al., 2006). After those reports showed an increased risk of breast cancer in women with HRT, the use of HRT decreased substantially worldwide (Maclennan et al., 2004).

As a result, the search for new therapies to overcome menopausal symptoms increased. As a result, complementary and alternative medicine, and nonhormone pharmaceuticals such as soy-derived products, St. John’s wort, and evening primrose oil gained more attention (Acog, 2001). However, none of these alternative medical treatments have been proven as being effective in treating vasomotor symptoms.

In East Asian countries, ginseng root has been widely used as an alternative medicine to improve general health. Ginsenoside is the major constituent of ginseng root and that in RG has been proven to have a wide range of biological and pharmacological activities (Kim et al., 2000). In a previous study, Kim et al. evaluated the effects of RG on menopausal symptoms and cardiovascular risk factors in postmenopausal women. There was a significant improvement in the Kupperman index and Menopause Rating Scale in the RG group compared to those of the placebo group (Kim et al., 2012). However, RG did not improve vasomotor symptoms. In contrast to women in Western countries, postmenopausal women in Asian countries report more joint pain than vasomotor symptoms (Mccarthy, 1994; Hilditch et al., 1999; Haines et al., 2005). Therefore, we evaluated the effect of RG on symptoms of degenerative OA in postmenopausal Asian women.

Previous *in vitro* and animal studies have shown the protective activity of RG against cartilage degradation (Kim et al., 2010; Endale et al., 2014; Lee et al., 2014; Lee et al., 2015). Some ginsenoside-enriched fractions inhibit both matrix metalloproteinase-13 (MMP-13) expression in IL-1β-treated human chondrocytes and the release of glycosaminoglycans from rabbit cartilage culture. MMP-13 is known to play an important role in the pathogenesis of OA by degrading type II collagens (Lee et al., 2015). In addition, red ginseng saponin extract has been shown to improve the severity of mouse collagen-induced arthritis (Kim et al., 2010). Ginsenosides including Rc, Rd, Rf, Rg1, Rg3, and F4 have inhibitory effects on MMP-13 expression in human chondrocytes. Ginsenosides Rg3 and F4 are contained only in RG (Endale et al., 2014; Lee et al., 2014). Based on these results, RG is believed to be effective in treating cartilage degradation-related disorders, but there has been no clinical study demonstrating this so far.

In this study, the pain score at various circumstances and DASH score improved with statistical significance in the RG group and the improvement in pain score at rest, during work or sport, and DASH score was significantly different compared to those of control group. Furthermore, the COMP level tended to decrease in the RG group after 12 weeks of treatment, and the change was statistically significant when compared to that in the placebo group, which showed an increase without significance. Serum COMP is one of the most consistent biomarkers associated with the diagnosis and prognosis of OA. Furthermore, serum COMP is influenced by various treatments and can be used as an indicator to evaluate the effectiveness of certain therapies in patients with OA (Tseng et al., 2009; Hosnijeh et al., 2015). Therefore, the COMP level results imply that RG can be a potential therapeutic option for OA in postmenopausal women.

A previous study reported that the activity of serum SOD was significantly increased after 12-weeks RG supplementation compared with the placebo group (Kim et al., 2012). In the present study, serum SOD levels were also significantly increased after 12-weeks RG supplementation, but these changes were not statistically significant compared with the placebo group. The discrepancy in results can be explained by the smaller sample size and greater age of participants, who were approximately 10 years older in the current study. Women experience OA more often after menopause than before. Women over 50 years of age experience estrogen deficiency after menopause and this may result in a higher prevalence and greater severity of cartilage degeneration of the joints. Therefore, a study with younger postmenopausal women is required to document the effects of age.

The limitations of this study include the small sample size and the single national background of the participants. Further studies on larger, more diverse ethnic populations are needed to apply the results to the general population. In addition, the pharmacokinetics of RG are not fully understood, so the mechanism for OA symptom improvement remains unknown. The side effects of RG are not well-studied yet. Even though RG is commonly accepted as a health supplement in Korea for people of all ages, its side effects include nervousness, insomnia, dizziness, and vaginal bleeding. Therefore, further investigations are needed to assess the safety and efficacy of RG in postmenopausal women.

To our knowledge, this is the first RCT to investigate the effect of RG on joint pain and function in postmenopausal women with degenerative OA. We also attempted to investigate whether biochemical markers of cartilage degradation were affected by RG. Given that previous studies have shown favorable effects of RG on antioxidative stress, RG may contribute to a decrease in pain and other degenerative OA symptoms as well as oxidative stress (Seo et al., 2014). In many OA patients, various anti-inflammatory analgesics are prescribed to relieve symptoms. However, gastrointestinal tract complications and bleeding are common side effects. Alternative medicines such as RG may present a brand-new, sustainable treatment option with fewer side effects for those suffering from degenerative OA.

## Data Availability

The original contributions presented in the study are included in the article/Supplementary Material, further inquiries can be directed to the corresponding authors.
