# Supplementary material for: Clinical Effects of Korean Red Ginseng in Postmenopausal Women With Hand Osteoarthritis: A Double-Blind, Randomized Controlled Trial
Source: Front Pharmacol. 2021 Nov 8;12:745568. doi: 10.3389/fphar.2021.745568 (PMC8630590; doi:10.3389/fphar.2021.745568)
Supplement: Supplementary file 1 [file DataSheet1.docx]

**HPLC analysis**

- The retention times of the sample

For the sample, the retention time for Rg1 was 5.563 minute, Rb1 was 14.096 minute, and Rg3 was 17.421 minute.
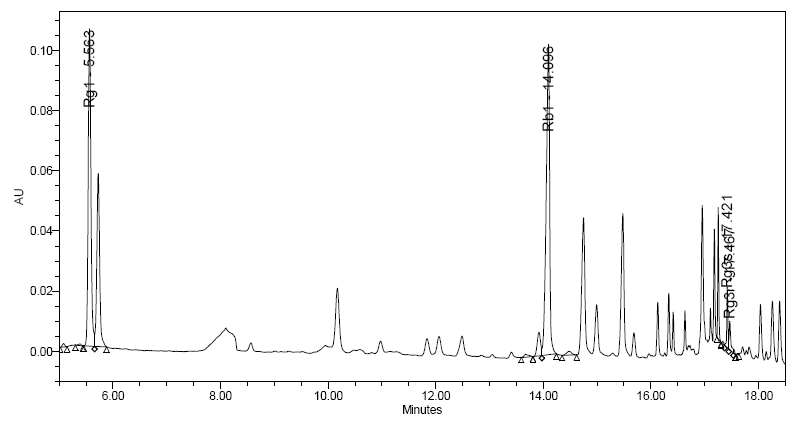


- The calibration curve concentration

The calibration curve concentration was as the following table.

| **Level** | | **Lv 1** | **Lv2** | **Lv3** | **Lv4** | **LV5** |
| --- | --- | --- | --- | --- | --- | --- |
| **Concentration (μg/mL)** | **Rg1** | 25.00 | 50.00 | 100.00 | 200.00 | 400.00 |
|  | **Rb1** | 31.25 | 62.50 | 125.00 | 250.00 | 500.00 |
|  | **Rg3s** | 6.25 | 12.50 | 25.00 | 50.00 | 100.00 |

- The lower limits of detection (LLOD) and lower limits of quantification (LLOQ)

The LLOD and LLOQ was as the following table.

| **Components** | **LOD(μg/mL)** | **LOQ(μg/mL)** |
| --- | --- | --- |
| **Rg1** | 1.74 | 5.80 |
| **Rb1** | 0.89 | 2.87 |
| **Rg3s** | 0.34 | 1.12 |

- Within run and between run precision

The data regarding precision was as the following tables.

**-Rg1**

| **Level of preparation** | **Level 1**  **(0.25g)** | **Level 2**  **(0.5g)** | **Level 3**  **(0.75g)** |
| --- | --- | --- | --- |
| **Mean (3 Replicate)** | 2.808 | 2.697 | 2.654 |
| **Standard deviation** | 0.009 | 0.073 | 0.007 |
| **Within-level precision**  **(%RSD)** | 0.33 | 2.70 | 0.25 |
| **Between-level mean** | 2.720 | | |
| **Between-level standard deviation** | 0.038 | | |
| **Between-level precision (%RSD)** | 1.38 | | |

**-Rb1**

| **Level of preparation** | **Level 1**  **(0.25g)** | **Level 2**  **(0.5g)** | **Level 3**  **(0.75g)** |
| --- | --- | --- | --- |
| **Mean (3 Replicate)** | 4.994 | 4.758 | 4.641 |
| **Standard deviation** | 0.020 | 0.123 | 0.012 |
| **Within-level precision**  **(%RSD)** | 0.39 | 4.65 | 0.26 |
| **Between-level mean** | 4.798 | | |
| **Between-level standard deviation** | 0.062 | | |
| **Between-level precision (%RSD)** | 1.30 | | |

**-Rg3s**

| **Level of preparation** | **Level 1**  **(0.25g)** | **Level 2**  **(0.5g)** | **Level 3**  **(0.75g)** |
| --- | --- | --- | --- |
| **Mean (3 Replicate)** | 0.228 | 0.211 | 0.211 |
| **Standard deviation** | 0.004 | 0.005 | 0.003 |
| **Within-level precision**  **(%RSD)** | 1.60 | 2.13 | 1.47 |
| **Between-level mean** | 0.217 | | |
| **Between-level standard deviation** | 0.001 | | |
| **Between-level precision (%RSD)** | 0.32 | | |

- Within run and between run accuracy

The data regarding accuracy and the percentage recovery was as the following tables.

**-Rg1**

| **Parameters** | **Rec.(100%)** | **Rec.(150%)** | **Rec.(200%)** |
| --- | --- | --- | --- |
| **Trial 1** | 100.2 | 100.9 | 101.4 |
| **Trial 2** | 100.2 | 102.6 | 101.5 |
| **Trial 3** | 100.0 | 101.9 | 100.8 |
| **Mean** | 100.0 | 101.9 | 100.8 |
| **Standard deviation** | 0.40 | 0.87 | 1.03 |
| **Precision (%RSD)** | 0.40 | 0.85 | 1.02 |

**-Rb1**

| **Parameters** | **Rec.(100%)** | **Rec.(150%)** | **Rec.(200%)** |
| --- | --- | --- | --- |
| **Trial 1** | 99.3 | 101.9 | 99.4 |
| **Trial 2** | 100.1 | 101.1 | 101.9 |
| **Trial 3** | 100.4 | 102.1 | 101.6 |
| **Mean** | 99.9 | 101.7 | 101.0 |
| **Standard deviation** | 0.55 | 0.51 | 1.40 |
| **Precision (%RSD)** | 0.55 | 0.50 | 1.39 |

**-Rg3s**

| **Parameters** | **Rec.(100%)** | **Rec.(150%)** | **Rec.(200%)** |
| --- | --- | --- | --- |
| **Trial 1** | 90.4 | 95.6 | 96.9 |
| **Trial 2** | 91.9 | 96.1 | 99.6 |
| **Trial 3** | 93.1 | 94.5 | 95.8 |
| **Mean** | 91.8 | 95.4 | 97.5 |
| **Standard deviation** | 1.36 | 0.82 | 1.96 |
| **Precision (%RSD)** | 1.49 | 0.86 | 2.01 |
